# Effects of Forest Management on the Insect Assemblage of Black Cherry (*Prunus serotina*) in the Allegheny National Forest

**DOI:** 10.3390/plants11192596

**Published:** 2022-10-01

**Authors:** Craig Larcenaire, Fumin Wang, Ida Holásková, Richard Turcotte, Michael Gutensohn, Yong-Lak Park

**Affiliations:** 1 USDA Forest Service, Forest Health Protection, Morgantown, WV 26505, USA; 2 Division of Plant and Soil Sciences, West Virginia University, Morgantown, WV 26506, USA; 3 Office of Statistics, West Virginia Agriculture and Forestry Experiment Station, West Virginia University, Morgantown, WV 26506, USA

**Keywords:** black cherry, shelterwood, silviculture, pollinators, pan traps, insect diversity

## Abstract

Over the last decade, the Allegheny National Forest (ANF) in the USA has experienced issues with the regeneration of black cherry (*Prunus serotina*). This study was conducted to investigate the effects of silvicultural treatment on the insect communities that may affect black cherry pollination and regeneration. We conducted a 2-year study to compare the abundance, richness, and diversity of insects in unmanaged, shelterwood seed-tree, and shelterwood clear-cut stands. Using pan traps, we sampled insects at the ground level and in the canopies of flowering mature black cherry trees. The results of this study showed significant increases in the abundance of insects captured in shelterwood seed-tree stands and in species richness and diversity of insects captured in the canopy of black cherry in shelterwood removal stands, indicating that silvicultural treatment affected the insect community significantly. The dominant insect order was Diptera (true flies, 72.91%, n = 12,668), and *Anthalia bulbosa* (Diptera: Hybotidae) was the dominant species comprising 33% of all insects found in the canopy of flowering black cherry. The findings in this study could help land managers in managing black cherry for its pollination and natural regeneration.

## 1. Introduction

The practice of managing forests has been used throughout history worldwide for economic and ecological purposes [1]. Black cherry (*Prunus serotina* Ehrh.) is an important tree species that is managed for its ecological and economic value in the northeastern USA [2]. The lumber is highly esteemed by woodworkers and carpenters, and the fruit is consumed by a wide diversity of wildlife [3]. Black cherry is widely distributed throughout the eastern USA and can grow in a variety of different climatic conditions. However, the highest quality black cherry timber originates from the Allegheny Plateau region of the USA where the environment is cool, moist, and temperate [3]. In particular, the Allegheny National Forest (ANF) located in northwestern Pennsylvania produces some of the highest-quality black cherry in the world. Over the last few decades, the land managers in the ANF have noticed a decline in natural regeneration. The cause of this decline is still unknown, though one hypothesis is a lack of adequate pollination and pollinators for the consequence of reduced fruit formation.

The management of timber species like black cherry requires the manipulation of a forest ecosystem using heavy machinery, which can have an impact on plant and animal communities including pollinators. Previous studies [4,5] showed that loss of tree habitat and soil erosion and compaction caused by silvicultural practices could have a lasting negative impact on some species of Lepidoptera (moths and butterflies). Other studies [6,7] suggest there is a positive response to open canopies or recently removed clear cuts by some solitary bee communities. However, when the dense herbaceous understory took over, the bee community’s richness and abundance were noticeably lowered [7]. These earlier studies indicate that the application of silvicultural management could affect insect abundance and diversity.

**Figure 1:**
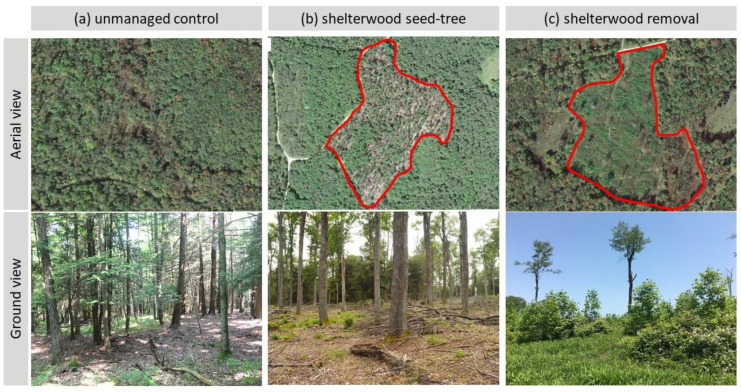
Aerial and ground views of unmanaged stand (**a**) and two different silvicultural management treatments (**b**,**c**) that are commonly used in the Allegheny National Forest located in Pennsylvania, USA. Red lines in (**b**,**c**) indicate the boundaries of management area. The shelterwood seed-tree treatment is conducted by removing approximately one-third of the overstory to encourage tree seedling development; mainly smaller trees and pole-sized understory trees are removed to provide at least 50 percent sunlight on the ground for seedling development. After approximately 3 to 15 years, the shelterwood removal treatment is conducted by removing the larger merchantable trees, typically leaving behind residual trees including den trees, snags, mast species, and uncommon species.

Currently, foresters in the ANF utilize even-aged regeneration treatments to manage shade-intolerant species like black cherry [8]. The preferred method for black cherry regeneration is the shelterwood seed tree cut (referred to as “seed-tree” hereafter) followed in succession by the shelterwood removal cut (referred to as “removal” hereafter). The seed-tree treatment is preferred where natural regeneration is desirable and when there is an opportunity on the landscape to increase management success [8]. This sequence of management involves harvesting approximately one-third of the overstory or mature trees originally found in the unmanaged stand (Figure 1a), leaving well-distributed trees that provide seeds for the next generation (Figure 1b). The seed-tree treatment provides sufficient light to reach the understory and promotes seedling establishment and growth, without giving enough light to undesired weeds. The removal cut is conducted approximately 3–15 years after the seed-tree treatment [8] (Figure 1c). The removal-cut proceeds when seedlings have been established, and a stand is at full maturity and ready to have all merchantable tree species harvested, leaving only the residual wildlife reserve trees [8]. This method is considered the best for the regeneration of Allegheny hardwoods [9]. Therefore, if there is not a significant quantity of black cherry saplings growing in the stands at the time of removal treatment, the potential for regeneration failure is significant. This failure is likely to occur because the canopy opening created by the removal treatment allows a new cohort of fast-growing herbaceous species to develop and out-compete any regenerating young black cherry trees. Additionally, a lack of viable seeds produced by the parent trees in the seed-tree stands could also be the cause of the black cherry regeneration problem in the ANF.

Only a couple of previous studies on black cherry pollinators have been published. Surveys conducted in a non-forest stand [10] and forest edge [11] reported that Hymenoptera (bees and wasps) and Diptera (true flies) are the most prevalent insect groups visiting black cherry flowers. Our previous study conducted in unmanaged black cherry stands [12] showed that the most abundant insects that visited black cherry canopies were insects belonging to the order Diptera followed by Coleoptera (beetles) and Hymenoptera. In addition, the study also found that insects, in particular dipterans, are attracted to the canopy of black cherry trees when flowers are present and thus likely contribute to pollination, and black cherry flowers emit a volatile blend that is similar to that of other pollinator-dependent *Prunus* species. However, little is currently known about how black cherry pollinators are affected by the management of respective forest stands, which can be a significant factor affecting the regeneration of black cherry.

To assist land managers in sustaining or even increasing black cherry populations, a further understanding of how different silvicultural systems affect the pollinator community is needed. Therefore, we conducted a 2-year field experiment to determine whether silvicultural treatments significantly affect the insect community in black cherry stands. Specifically, our study used pan traps installed in the canopy and on the grounds to capture insects visiting black cherry during flowering to determine if two silvicultural treatments (i.e., seed-tree and removal treatments) increase or decrease the species abundance, richness, and diversity (see details in Section 4) of insects compared to unmanaged black cherry trees (i.e., control). We hypothesized that the two silvicultural treatments would reduce the abundance, richness, and diversity of insect species visiting black cherry flowers. We further hypothesized that insects utilizing and pollinating black cherry flowers would be found in the stands with the highest concentration of black cherry (i.e., seed-tree treatment).

## 2. Results

### 2.1. Overall Abundance of Insects Captured

Over the two-year trapping period using pan traps in the canopy and on the ground at three different stages of silvicultural treatments (i.e., unmanaged control stands, seed-tree stands, and removal stands), 17,375 insects were collected (see summary in Table 1). The major insect orders captured were Diptera (true flies, 72.91%, n = 12,668), Coleoptera (beetles, 10.24%, n = 1780), Hymenoptera (bees and wasps, 8.51%, n = 1478), Lepidoptera (moths and butterflies, 3.86%, n = 670), and Thysanoptera (thrips, 3.52%, n = 612). All other insect orders only comprised <1% of the total capture (i.e., Collembola, Mecoptera, Orthoptera, Plecoptera, and Psocoptera, n = 167). With all the insect orders combined, insect captures were the highest in the seed-tree stands (n = 8712, 50.14%), followed by removal (n = 4771, 27.46%) and control stands (n = 3892, 22.40%). The canopy and ground traps accounted for 59.91% (n = 10,409) and 40.09% (n = 6966) of total captures, respectively. However, the relative gross proportions (unadjusted to other experimental design factors) of individual insect orders were not only associated with the three silvicultural treatments (χ^2^ = 595.24, *p* < 0.001), but also with the two trap positions (χ^2^ = 345.17, *p* < 0.001).

### 2.2. Effects of Silvicultural Practice on Insect Abundance

**Figure 2:**
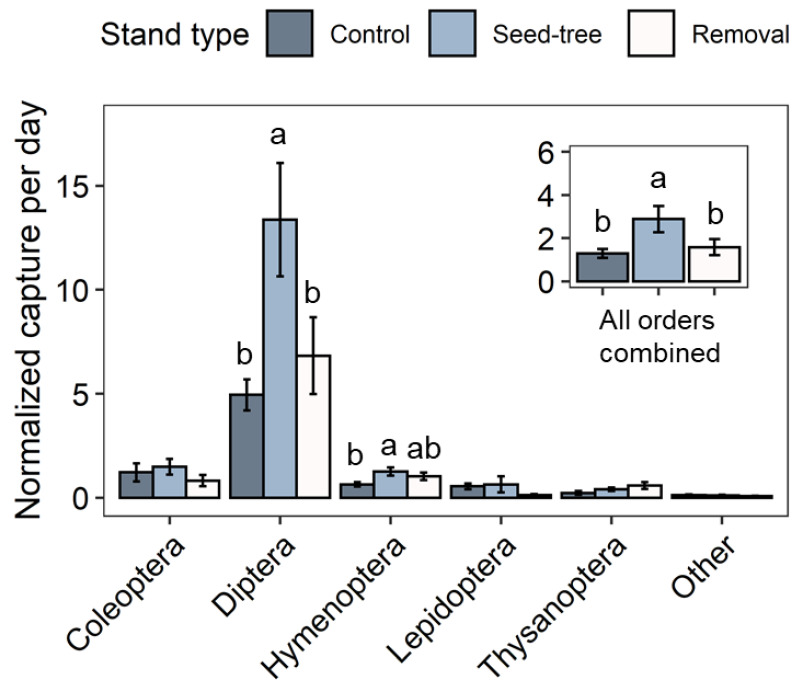
The abundance of insect orders captured under different silvicultural practices (control, seed-tree, and removal treatments) during the black cherry flowering period. The significant effects of stand type on overall abundance (insert, *p* < 0.001), Diptera (*p* = 0.001), and Hymenoptera (*p* = 0.016) were detected in repeated measures ANOVA. The least-square means of these groups were compared using the Tukey–Kramer test with adjustment; groups with the same letter are not significantly different at α = 0.05.

Utilizing repeated measures ANOVA, we found a significant effect of silvicultural treatment on the captures of all insect orders combined (df = 2, F = 7.94, *p* = 0.001; Figure 2). Specifically, higher trap captures were observed in the seed-tree stands when compared to the removal (df = 66, t = −3.41, Adj. *p* = 0.003) and control (df = 66, t = −3.49, Adj. *p* = 0.003) stands. The effect of silvicultural treatment was also found to be significant on the abundance of Diptera (df = 2, F = 7.74, *p* = 0.001) and Hymenoptera (df = 2, F = 4.44, *p* = 0.016). In particular, Diptera were significantly more abundant in the seed-tree stands than in control stands (df = 66, t = −3.59, Adj. *p* = 0.002) and removal stands (df = 66, t = −3.19, Adj. *p* = 0.006) (Figure 2). Similarly, the abundance of Hymenoptera was significantly higher in the seed-tree stands than in control stands (df = 66, t = −2.94, Adj. *p* = 0.012) (Figure 2). These results indicate that seed-tree treatments increased the overall abundance of insects including two major insect orders (i.e., Diptera and Hymenoptera).

### 2.3. Relationship between Trap Position and Insect Abundance

**Figure 3:**
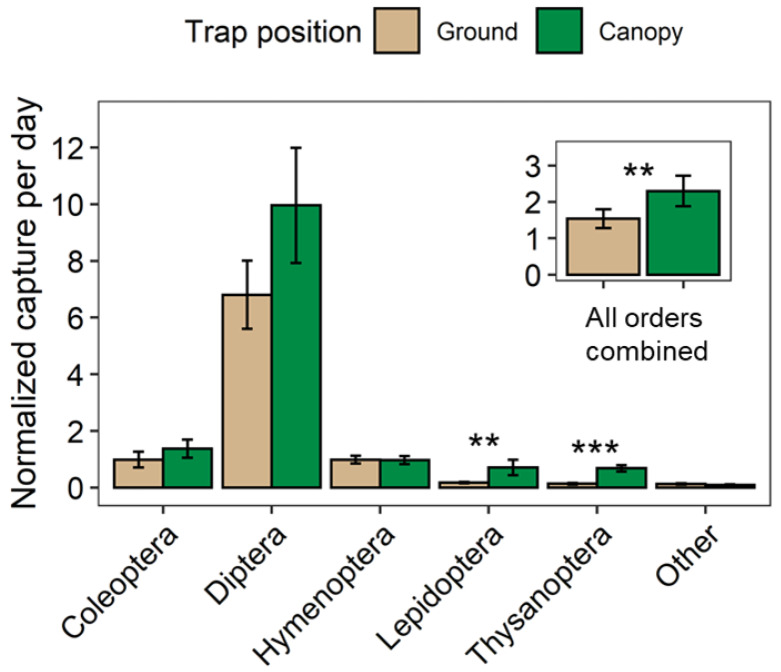
The abundance of insects captured in traps placed on the ground and in the canopy during the blooming period of black cherry. Asterisks indicate a significant effect of trap position on abundance of specific insect orders (**, *p* < 0.01; ***, *p* < 0.001).

Trap position showed a significant (df = 1, F = 8.85, *p* = 0.004) effect on the captures of all insect orders. Specifically, more insects were captured in the canopy than on the ground (Figure 3, insert). The main effect of trap position on individual orders was significant in Lepidoptera (df = 1, F = 9.61, *p* = 0.003) and Thysanoptera (df = 1, F = 41.81, *p* < 0.001). Both orders showed a higher abundance in the canopy traps than in the ground traps (Figure 3).

### 2.4. Effects of Silvicultural Treatment and Trap Position on the Species Richness of Insects

**Figure 4:**
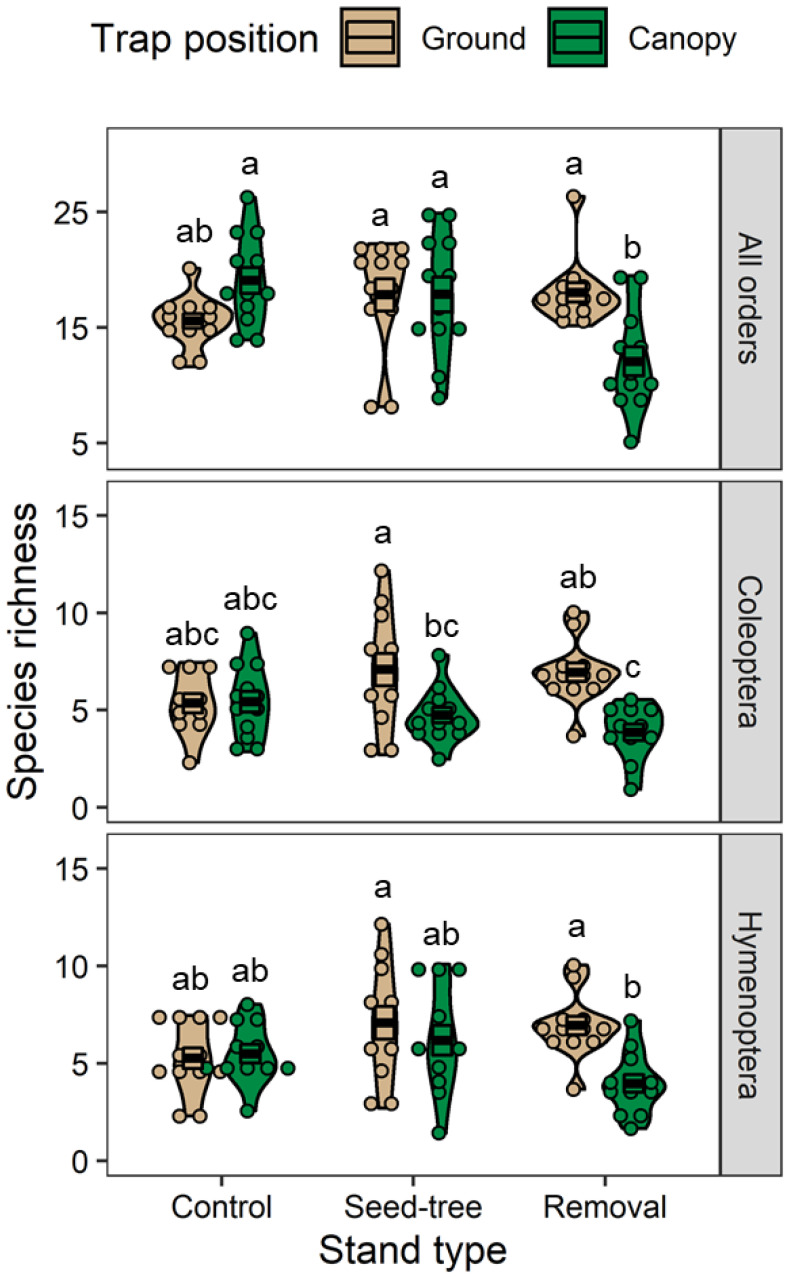
Crossbars (mean ± SE) and violin plots (probability density), including individual measures (n = 12 traps per group) of species richness for all insect orders combined, Coleoptera, and Hymenoptera. Values in each panel are compared due to the significant interaction between trap position and stand type. Groups with different letters indicate significant differences (*p* < 0.05, Tukey–Kramer test).

We identified 216 morphospecies in Diptera, 172 in Hymenoptera, 99 in Coleoptera, 55 in Lepidoptera, and 2 in Thysanoptera. Silvicultural treatment significantly affected the species richness of all orders combined (df = 2, F = 3.22, *p* = 0.046), and its interaction with trap position was also significant (df = 2, F = 8.47, *p* < 0.001). The species richness in the removal stands was significantly higher in ground traps than in canopy traps (df = 66, t = 3.62, Adj. *p* = 0.007), whereas that in control and the seed-tree stands did not differ between ground and canopy traps (Adj. *p* > 0.05; Figure 4).

We found that, for Coleoptera and Hymenoptera only, the insect richness was significantly affected by trap position (Coleoptera: df = 1, F = 15.43, *p* < 0.001; Hymenoptera: df = 1, F = 5.92, *p* = 0.018) (Figure 4) and its interaction with silvicultural treatment (Coleoptera: df = 2, F = 4.31, *p* = 0.018; Hymenoptera: df = 2, F = 3.50, *p* = 0.036). Insect richness was not significantly affected by silvicultural treatment or trapping position in remaining insect orders (Adj. *p* > 0.05). For Coleoptera, the species richness in the removal and seed-tree stands were both significantly higher in ground traps than in canopy traps (removal stands: df = 63, t = 3.93, Adj. *p* = 0.003; seed-tree stands: df = 63, t = 3.05, Adj. *p* = 0.037), while that in control stands did not differ between ground and canopy (Adj. *p* > 0.990). For Hymenoptera, the species richness in the removal stands was significantly higher in ground traps than in canopy traps (df = 65, t = 3.46, Adj. *p* = 0.012), whereas that in both control and seed-tree stands did not differ between ground and canopy traps (Adj. *p* > 0.05; Figure 4).

### 2.5. Effects of Silvicultural Treatment and Trap Position on the Species Diversity of Insects

**Figure 5:**
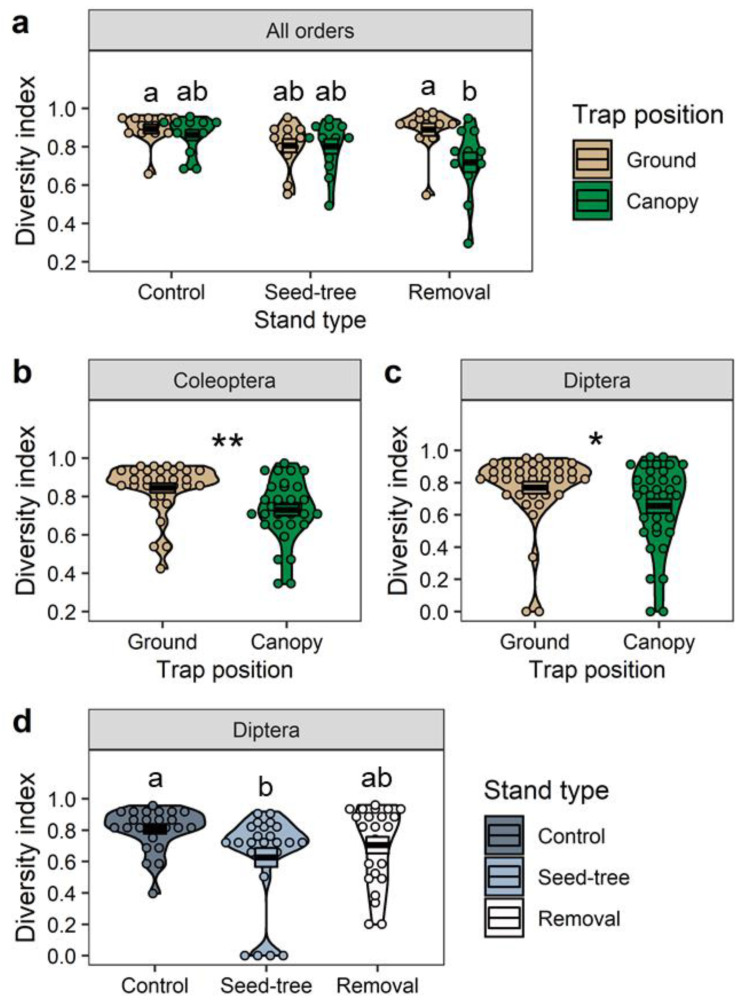
Crossbars (mean ± SE) and violin plots (probability density) of Simpson’s Index of Diversity. The significant interaction between trap position and stand type for all insect orders combined (**a**; *p* = 0.031), the significant main effect of trap position on Coleoptera (**b**; **, *p* < 0.01) and Diptera (**c**; *, *p* < 0.05), and the significant main effect of stand type on Diptera (**d**; *p* = 0.032). Mean values within panels (**a**,**d**) were compared using the Tukey–Kramer test with adjustment; groups with the same letter are not significantly different at α = 0.05.

The Simpson’s Index of Diversity (1-D) was used here as a measure of species diversity with the range of 0–1; it increases as species diversity increases. We found a significant interaction between the effects of trap position and silvicultural treatment (df = 2, F = 3.66, Adj. *p* = 0.031) on the diversity of all insect orders combined. Specifically, the species diversity of all the insects captured in the removal stands was significantly higher in the ground traps than in canopy traps (df = 66, t = 3.67, Adj. *p* = 0.006), whereas that in the control and seed-tree stands did not differ between ground and canopy traps (Adj. *p* > 0.05; Figure 5a). For Coleoptera, the effect of trap position was significant, with the diversity being significantly lower in canopy traps than ground traps in all three stand treatments combined (df = 1, F = 11.25, *p* = 0.001; Figure 5b). For Diptera, both trap position (df = 1, F = 4.80, *p* = 0.032) and stand type (df = 2, F = 3.79, *p* = 0.028) showed significant main effects. The diversity of the order Diptera was significantly lower in canopy traps than ground traps (Figure 5c) in all three stand treatments combined. Simultaneously, the diversity of Diptera in the control stands was significantly higher than that in seed-tree stands (df = 65, t = 2.75, Adj. *p* = 0.021), but not significantly higher than that in the removal stands (df = 65, t = 1.37, Adj. *p* = 0.361; Figure 5d).

### 2.6. Effects of Silvicultural Treatment and Trap Position on the Abundance of Major Insect Species

We found 547 morphospecies from the 2-year trapping in this study. The top ten most abundant insect species found in this study accounted for 69.47% of the total capture (see a list in Table 2). They include *Anthalia bulbosa* (Diptera: Hybotidae, 32.78%), *Smittia* spp. (Diptera: Chironomidae, 13.18%), *Rhamphomyia* spp. (Diptera: Empididae, 5.46%), *Byturus unicolor* (Coleoptera: Byturidae, 4.22%), *Frankliniella* spp. (Thysanoptera: Thripidae, 3.51%), *Discocerina* spp. (Diptera: Ephydridae, 2.98%), *Omalium* spp. (Coleoptera: Staphylinidae, 2.02%), *Cynipini* (Hymenoptera: Cynipidae, 1.81%), *Melanolophia canadaria* (Lepidoptera: Geometridae, 1.76%), and *Rhagio mystaceus* (Diptera: Rhagionidae, 1.76%). Due to the strong sexual dimorphism, *A. bulbosa* was initially counted as two separate morphospecies, which accounted for 20.20% (male) and 12.58% (female) of total capture, respectively.

Silvicultural treatment affected the abundance of some of these major insect species observed in this study. According to rank-based nonparametric multiple comparisons, *Smittia* spp. was significantly more abundant in the seed-tree stands than in removal and control stands (Dunn test, Adj. *p* < 0.05; Table 2). *Frankliniella* spp. was significantly more abundant in the removal and control stands, while *Omalium* spp. was significantly more abundant in the seed-tree and control stands (Dunn test, Adj. *p* < 0.05; Table 2). On the other hand, female *A. bulbosa* as well as four other species (i.e., *Smittia* spp., *Frankliniella* spp., Cynipini, and *M. canadaria*) were more abundant in the canopy traps than in the ground traps (Wilcoxon rank-sum test, *p* < 0.05; Table 2). Reversely, both B. unicolor and R. mystaceus were more abundant in the ground traps compared to the canopy traps (Wilcoxon rank-sum test, *p* < 0.05; Table 2).

## 3. Discussion

Black cherry is an economically and environmentally important species to the Allegheny Plateau region of the USA, and silvicultural management of the species is necessary for timber production. The goal of this study was to examine if there was a potential deficiency in the insect communities in black cherry stands during the flowering period and if forest silvicultural treatments influence the species diversity and richness of the insects associated with black cherry. Our results showed that the major insect order was Diptera regardless of stand type and trap position, comprising ~73% of 17,375 insects captured in this study (Table 1). Many insects collected in this study are known to feed on pollen or are associated with flowers. Our previous study [12] via electron microscopy analysis demonstrated that insects collected in the canopy traps, representing 12 different families in three major orders (Coleoptera, Diptera, and Hymenoptera), carried pollen grains of black cherry on their bodies, legs, and antennae. These insects included *Antocha* sp. (Diptera: Limoniidae), *Atalantycha bilineata* (Coleoptera: Cantharidae), *Camponotus pennsylvanicus* (Hymenoptera: Formicidae), *Trichopion* sp. (Coleoptera: Curculionidae) and *Drosophila* sp. (Diptera: Drosophilidae).

Our study also revealed that the canopy traps captured significantly more insects (~60% of all insects captured in this study) than the ground traps (Table 1, Figure 3). In line with our previous study [12] two specific insect orders (i.e., Lepidoptera and Thysanoptera) were found to be significantly more abundant in the canopy of black cherry, indicating that these insects might be utilizing flowers and thus contributing to the pollination of black cherry. Likewise, the number of Diptera captured in the canopy of black cherry trees was higher than that on the ground (Table 1), however, in contrast to our previous study [12], the difference in Diptera captured in the two trap positions was not significant (*p* = 0.123) in the current study (Figure 3). Remarkably, the species diversity of all the insect species and that of Diptera in the removal treatment were significantly higher in the ground traps than in canopy traps (Figure 5), suggesting that the resources (i.e., nectar and pollen) in the removal stands were less abundant in the canopy than on the ground. The lack of contiguous canopy cover for the insect to move within might also contribute to the decreased number of insects found in the canopies in removal stands. Although many dipteran insects are predators (such as syrphid larvae) or associated with pasture or livestock, we did not find such dipteran species in our study because it was conducted inside the forest distant from field crops and livestock farms. In addition, honeydew produced by sap-sucking insects such as aphids can attract other insects including Coleoptera and Diptera. As the flowering period of black cherry is quite early in spring, we did not observe a high number of aphids at the time of black cherry flowering. Additionally, we did not find or recognize any sooty mold growing on lower leaves which might have been caused by a high abundance of honeydew-producing insects.

A mature black cherry in the ANF can grow to heights of up to 35 m, which is a significant distance for a small flower-visiting insect living on the ground to fly upward to find a source of pollen and nectar in the canopy. Therefore, we believe that most of the insects we found in the canopy during flowering were attracted to the flowers and voluntarily flew up to the canopy. In addition, significantly higher overall insect abundance was observed in the seed-tree stands, suggesting that the seed-tree treatment created a resource-abundant environment that attracted these species to the canopy of the remaining flowering black cherry trees. The resource concentration hypothesis predicts that if there is a resource abundance it will support higher loads of plant-specific species [13]. Conversely, resource dilution will not support plant-specific insect species and thus their number will be reduced [14]. In our study, the seed-tree stands had a higher concentration of resources for black cherry-specific flower visitors, which could explain why the flower-visiting insects including Dipteran were more abundant where black cherry trees were in higher concentrations. This can be seen in the control stand where black cherry comprises <3% of the total tree species. Conversely, in the seed-tree stands where we observed a higher percentage of black cherry (~30%), we also observed a higher population of insects. Furthermore, when the resources are removed in the next step of the treatments (i.e., removal treatment), we observed a drop in the insect population back to low black cherry concentration conditions.

The seed-tree treatment removes approximately one-third of the competing overstory and understory tree species in a forest stand (Figure 1), which lowers the tree diversity in the stand in favor of mature black cherry in the overstory. Some insects in the Diptera and Lepidoptera orders that indeed are associated with and use black cherry flowers, were concentrated in the canopy of these remaining seed-tree stands (Table 2). Many lepidopterans we found in this study are polyphagous and can feed on the vegetation of many different tree and plant species as larvae. In contrast, the significantly lower number of dipterans and lepidopterans found in the removal stands could be explained by the substantial loss of overstory canopy structure caused by the clearcutting or removal of most black cherry trees and in consequence the disturbance of habitats. This treatment is designed to drastically lower the number of trees per ha and tree diversity in a stand by harvesting all merchantable tree species. This creates large openings that decrease humidity, increase sunlight to the understory, and raise windspeeds in the stand. These conditions, along with the temporary reduction in the diversity of floral resources, are less favorable for flower-visiting insects. We conducted ground surveys of the understory herbaceous vegetation during the peak flowering period of black cherry. From the understory vegetation survey, we found 39 plant species in the control, 22 species in the seed-tree cut, and 18 species in the removal treatments (Table S1).

Our study revealed that seed-tree treatments increased insect species abundance in two major insect orders (i.e., Diptera and Hymenoptera) and species richness was significantly affected by trap position in two insect orders (i.e., Coleoptera and Hymenoptera). The only order to show an interaction of stand type and trap position was Lepidoptera (data not shown). From our observations, we have seen that lepidopterans prefer diverse vegetation and inhabit the canopies of trees. These results indicate that the captures of lepidopterans depend on silvicultural treatments and the location of insect traps. If the stand has more diverse overstory vegetation higher numbers of lepidopterans are expected to be captured in the canopies.

A total of 544 morphospecies in the five major insect orders (i.e., Diptera, Coleoptera, Lepidoptera, Hymenoptera, and Thysanoptera) were found in this study, and the seed-tree treatment significantly affected the species abundance, richness, and diversity. In the seed-tree and control stands, lepidopterans were more abundant in the canopy, suggesting that lepidopterans prefer a stand with more diverse and abundant overstory; these insects are likely using the canopy to feed as larvae and as a source of nectar as adults. The lepidopteran species most frequently found in the canopy was *M. canadaria*. This is a polyphagous insect that is known to feed on the leaves of nearly every deciduous tree species found in the control and seed-tree stands as a larva [15]. The polyphagous adults have also been recorded to carry black cherry pollen on their bodies which indicates they use black cherry by feeding on nectar and likely contribute to pollination [16]. Notably, our study showed the scarcity of hymenopterans found inside the forest, compared to a previous study conducted on the forest edge [11] that reported a high number of hymenopterans. Major hymenopteran species found in our study was gall-inducing wasps which are known to be associated with oak, but their ecological importance to black cherry pollination is not known.

The main species of thrips captured in the canopy was identified as *Frankliniella occidentalis* (Thysanoptera: Thripidae), western flower thrips. *F. occidentalis* is known as a flower dweller and adults live amongst the foliage, fruits, and flowers of a wide variety of plant species [17]. The larvae typically drop to the ground when they are ready to pupate but can remain on the plant if the floral structure is complex enough [16]. Since the 1970s *F. occidentalis* has spread from its native range in western North America to become a major pest of horticultural and agricultural crops worldwide [18]. It is believed that this species arrived in the area through infested plants being transported to greenhouses in Pennsylvania [18]. These are important pests economically due to their feeding habits on flowers (florivore) and their ability to transmit viruses [19]. Our previous study [12] found that some of the volatile organic compounds (VOCs) emitted by the flowers of black cherry match VOCs that have been demonstrated to be attractive to *F. occidentalis* [20]. *Prunus* necrotic ringspot virus (PNRSV), and prune dwarf virus (PDV) can be transmitted through pollen found on the bodies of thrips and can cause up to 50% yield loss in sweet cherry and 100% yield loss in peach [21,22]. Although these viruses have not been confirmed in black cherry, there is the potential for a negative impact on black cherry regeneration.

Based on our findings, a potential reason for the decline in the natural regeneration of black cherry observed by land managers in the ANF could be a lack of adequate pollination of the flowers. One might expect that the increased abundance of all insects upon the seed-tree treatment (Table 1, Figure 2) and in particular of Diptera, which we had found previously to carry black cherry pollen [14], would result in an improved pollination rate. However, pollination in black cherry, like in other *Prunus* species, is controlled by a self-incompatibility mechanism [23,24] which is genetically regulated by the multi-allelic S-locus. Thus, for successful pollination black cherry requires the transfer of pollen from flowers of a tree with one set of S-locus alleles to the flowers of another tree carrying a distinct set of S-locus alleles to produce viable seeds. In contrast, when the transferred pollen and the pistil of the receiving flower express the same S-locus allele the pollen is rejected which results in a failure to fertilize the flower as well as to form seeds and a fruit. The gradual reduction in the number of black cherry trees in the seed-tree and removal treatments concomitantly increases the distance between individual black cherry trees. Many insects visiting flowering black cherry trees are relatively small in size and therefore have a weak ability to fly suggesting that an increased distance between trees after these silvicultural treatments might actually reduce the rate of required cross-pollination. Moreover, the removal of trees in forest stands likely also leads to a reduced representation of diverse S-locus alleles in the black cherry population, which in consequence might increase the chance of pollen rejection due to the flower pistil expressing the same S-allele. Further studies will be required to verify if the silvicultural treatments currently used in the ANF not only alter the insect assemblage associated with black cherry but also create a potential genetic bottleneck in the tree population which could contribute to the decline in natural regeneration.

In this study, we examined how the insect community visiting black cherry trees would be influenced by silvicultural management. We hypothesized that the two silvicultural treatments (i.e., shelterwood seed-tree and removal treatments) would reduce the abundance, richness, and diversity of insect species visiting black cherry flowers. The results of our experiment showed that the seed-tree treatment increases the overall abundance of insects visiting black cherry during the flowering period, and the species richness and diversity of insects captured in the canopy of black cherry were lower in removal treatments, indicating that silvicultural treatments affected insect community significantly. In addition, our study showed that Diptera was the most abundant insect order, and we listed 10 major insect species visiting flowers of black cherry in the seed-tree and removal treatments, suggesting they are key players in the pollination of black cherry. Furthermore, our previous study [12] showed a significant increase in dipteran species when black cherry is flowering. The current study revealed that the concentration of black cherry in a managed forest stand also has an effect on dipteran abundance, further providing evidence that dipterans are attracted by the flowers of black cherry and are potentially significant pollinators.

Although our study reported various aspects of insect community responses to the silvicultural treatments in black cherry stands, future studies are needed to investigate if the structural changes in the canopy resulting from silvicultural treatments can impact the movement of pollinator insects between trees, which is required to overcome self-incompatibility issues in black cherry pollination. In addition, further studies should investigate whether the major insects we captured are efficiently cross-pollinating black cherry trees. As the silvicultural practices presented in this manuscript are successive interventions, a long-term study including both practices would provide a more comprehensive understanding of their effect on pollinator insects.

## 4. Materials and Methods

### 4.1. Study Sites and Experimental Design

This study was conducted at two sites located in the Bradford Ranger District in the ANF. The first site was located in Cherry Grove (41°43′15.4″ N 79°07′16.7″ W) in Warren County, while the second site was in McKean County near the city of Bradford (41°44′04.2″ N 78°41′59.1″ W). Each of the selected sites was approximately 12 ha and adjacent to a forest service road. At each site, three plots with different stages of silvicultural treatments (i.e., unmanaged control, seed-tree, and removal) were assigned. The plots were delineated using satellite imagery and ArcGIS (ESRI, Redland, CA, USA) to define boundaries (Figure 1). The two sites were approximately 35 km apart, and any two plots in each site were approximately 1 km apart. The seed-tree and removal treatments in Bradford were applied in 2015 and 2017, respectively, while both treatments were applied in 2015 at Cherry Grove. All control plots are essentially mixed-aged hardwood stands consisting of typical upland hardwood species such as eastern hemlock (*Tsuga canadensis*), American beech (*Fagus grandifolia*), tulip poplar (*Liriodendron tulipifera*), maple (*Acer* spp.), birch (*Betula* spp.), and black cherry.

The experimental arrangement of treatments was a split-plot design with main plots in randomized blocks. This kind of design is applicable when experimental units (EU) of different sizes are utilized. The three stages of silvicultural treatments represented the main plot (i.e., control, seed-tree, and removal), where the EU was a whole tree with three replications (i.e., trees), considered independent of each other. The two locations (Cherry Grove and Bradford) served as random blocks since all of the treatment combinations (treatment and trap positions) were sampled at the same time at each location. The split-plot factor was the trap position (i.e., on the ground and in the canopy), where the EU is a portion of a tree. Within each treatment by position combination, we chose three mature, overstory black cherry trees using a random point generator in ArcGIS. The random points map was uploaded to a Juno^®^ GPS unit (Trimble Inc., Sunnyvale, CA, USA), and a random number generator dictated which point was chosen. The closest black cherry tree to the point on the map was chosen. This arrangement provided a total of 36 trapping arrays (2 locations x 3 treatments x 2 positions x 3 replications or trees) to be collected and compared over two seasons (years) during the same seven-day period of black cherry trees blossoming.

### 4.2. Insect Sampling

To sample insects visiting black cherry, a trapping array consisting of three different colored (white, yellow, and blue) pan traps were deployed at both ground and canopy levels of each sampled tree [12]. These pan traps were made from white 355-mL plastic cups (Solo, Lake Forest, IL, USA). Fluorescent yellow and fluorescent blue were painted with fluorescent pigmented paint (Fluorescent Blue and Yellow dispersion, Guerra Paint and Pigment Corp, New York, NY, USA) mixed with a water-based matte flexible acrylic polymer emulsion (Silica Flat, Guerra Paint and Pigment Corp., New York, NY, USA). A solution of ~25-mL unscented dish soap (Free and Clear dish soap, Seventh Generation, Burlington, VT) per one gallon of water was used to fill the cups. This solution is used to break the surface tension to trap the insects that land in the cup. These colors and the trap design have been demonstrated to attract different orders of insects [25,26]. The cups were affixed to a platform made from a 22.7-L bucket lid with three 7.6 cm holes. A 3-pronged metal hanger was attached to the platform for added rigidity and tie points. The canopy traps were hung in the selected tree crown using a slingshot (Big Shot Slingshot, Sherrill Tree, Greensboro, NC, USA) and paracord rope [12]. These traps were hung approximately halfway into the canopy near the flowers approximately 20 m above the ground. The ground traps were placed near the base of the tree trunk on a 30 cm wooden stake which is attached to a bucket lid. All traps were deployed for seven days during the peak flowering of black cherry, and the field experiment was repeated at the same trees in 2018 and 2019.

The insects captured in the traps were strained using a fine mesh paint strainer (Mesh Paint Strainer, Harbor Freight Tools, Camarillo, CA, USA) and stored in a sample bag (Whirl Pak, Nasco, Atlanta, GA, USA) with 70% ethyl alcohol. The sample bag was labeled with the trap identification number, date, and trap color. The samples were stored in vials with 70% ethanol and identified to order and morphospecies [27] by using a digital microscope (Olympus SZ71 microscope and Olympus DP21, Cell Sens Dimension, Olympus Inc., Tokyo, Japan). Major insect species found in the samples were further identified to species with the help of insect taxonomists: Robert Acciavatti (Coleoptera), Andrea Kautz (Diptera), Sam Droege (Hymenoptera), and Gwan-Seok Lee (Thysanoptera).

### 4.3. Statistical Analysis

The total numbers of each insect order (i.e., Coleoptera, Diptera, Lepidoptera, Hymenoptera, Thysanoptera, and other insect orders) captured throughout the two trapping years were calculated by each trap position from each silvicultural treatment, while the association between insect order and silvicultural treatment and that between insect order and trap position were analyzed by Pearson’s Chi-square frequency tests.

Treatment structure for statistical modeling included the fixed effects of treatment, trap position, and their interaction. Random effects were the two sites and sites by treatments to represent the whole plot error. Insect order was deemed as a random effect in statistical model when all orders were combined, and the response variable was the total insect count across all orders. Repeated effects were the year (2018 and 2019) and trap position, using trap as a subject (i.e., experimental unit) in doubly repeated measures ANOVA. Prior to modeling, the total trap captures of each insect order were combined from each trapping array [12] and the sums were divided by trapping days to get normalized captures, the response variable. Proc GLIMMIX (generalized linear mixed model) of SAS with estimation technique restricted maximum likelihood and Kenward-Roger denominator degrees of freedom method were utilized. Normality of model residuals was examined by Shapiro–Wilk W-Test [28], and a square root transformation (i.e., $\sqrt{count+0.0001}$) was used to compensate for the lack of normality when necessary. Tukey’s HSD was used to adjust *p*-values for multiple comparisons of least square means.

In addition, the richness and diversity (i.e., Simpson’s Diversity Index) of each insect order (Coleoptera, Diptera, Lepidoptera, and Hymenoptera) and that of all orders combined were calculated [29] by each trap position from each treatment based on morphospecies. Species richness was calculated as (s−1)/logN, where s is the number of species and N is the total number of individuals in all species. The Simpson’s Diversity Index was calculated as $1-\sum \frac{n\left(n-1\right)}{N\left(N-1\right)}$ where n is the number of individuals for each species and N is the total number of all individuals. Thysanoptera was excluded from the calculation of individual diversity and richness indices since it contained only two distinct morphospecies but included for calculating the indices for all orders combined. The Simpson’s Diversity indices were arcsine-transformed to improve residual normality and GLIMMIX model as above was used. Tukey’s HSD was used to adjust p-values for multiple comparisons of least square means.

Counts of individual morphospecies were summarized by treatment and trap position. The effect of treatments on individual morphospecies was examined by Kruskal–Wallis test with Chi-Square approximation, and Dunn test was used for pairwise comparison with *p*-values adjusted by Benjamini-Hochberg procedure. The effect of trap position was assessed by Wilcoxon rank-sum test with continuity correction. All statistical analyses were performed in JMP^®^ Pro 16.0 and SAS^®^ 9.4 (SAS Institute Inc., Cary, NC, USA) at ⍺ = 0.05.

## Figures and Tables

**Table 1:** The abundance of insects from different orders collected by two-year trapping in three different stand types of forest management (i.e., control, seed-tree, and removal) at two different trap positions (i.e., ground and canopy).

Stand	Position	Diptera	Coleoptera	Hymenoptera	Lepidoptera	Thysanoptera	Other Orders	All Orders	All Orders %
Control	Ground	771	333	131	63	17	44	1359	7.82
Control	Canopy	1721	282	194	213	101	22	2533	14.58
Seed-tree	Ground	2796	181	298	37	37	30	3379	19.45
Seed-tree	Canopy	3942	570	336	288	168	29	5333	30.69
Removal	Ground	1577	231	317	33	48	22	2228	12.82
Removal	Canopy	1861	183	202	36	241	20	2543	14.64
Ground and canopy traps combined									
Control stands		2492	615	325	276	118	66	3892	22.40
Seed-tree stands		6738	751	634	325	205	59	8712	50.14
Removal stands		3438	414	519	69	289	42	4771	27.46
All stands combined									
Ground traps		5144	745	746	133	102	96	6966	40.09
Canopy traps		7524	1035	732	537	510	71	10,409	59.91
Total		12,668	1780	1478	670	612	167	17,375	-
Total %		72.91	10.24	8.51	3.86	3.52	0.96	-	-

**Table 2:** List of the 11 most abundant morphospecies (10 species) captured in different stand types of forest management (control, seed-tree, and removal) at two positions (ground and canopy). Captures of each morphospecies by the three treatments (control, removal, and seed-tree) were analyzed by Kruskal–Wallis test with Chi-Square approximation. Dunn test was used for pairwise comparison with *p*-values adjusted by Benjamini-Hochberg procedure, while different letters within species indicate significant differences among stand types (Adj. *p* < 0.05). Captures by the two trap positions were analyzed by Wilcoxon rank-sum test with continuity correction (*, *p* < 0.05; **, *p* < 0.01; ***, *p* < 0.001).

Order	Family	Tribe/Genus/Species	Total	Total %	Stand Type			χ^2^ (df = 2)	*p*-Value	Trap Position		*W* (df = 1)	*p*-Value
					Control	Seed-Tree	Removal			Ground	Canopy		
Diptera	Hybotidae	*Anthalia bulbosa* (♂)	3412	20.20	513	1553	1346	1.95	0.378	1839	1573	3291	0.439
	Hybotidae	*Anthalia bulbosa* (♀)	2126	12.58	310	1072	744	2.83	0.243	38	2088	1176	<0.0001 ***
	Chironomidae	*Smittia* spp.	2226	13.18	579b	1413a	234b	19.82	<0.0001 ***	875	1351	6688	0.785
	Empididae	*Rhamphomyia* spp.	922	5.46	195	533	194	0.27	0.874	497	425	3406.5	0.259
	Ephydridae	*Discocerina* spp.	503	2.98	119	324	60	5.04	0.080	94	409	1290	0.190
	Rhagionidae	*Rhagio mystaceus*	297	1.76	37	180	80	1.22	0.545	229	68	543.5	0.045 *
Coleoptera	Byturidae	*Byturus unicolor*	713	4.22	309	175	229	1.68	0.433	540	173	1018.5	0.012 *
	Staphylinidae	*Omalium* spp.	341	2.02	32b	241a	68ab	8.00	0.018 *	31	310	449.5	0.071
Thysanoptera	Thripidae	*Frankliniella* spp.	593	3.51	113b	192ab	288a	7.25	0.027 *	97	496	2243	<0.0001 ***
Hymenoptera	Cynipidae	Cynipini	306	1.81	57	153	96	4.81	0.090	13	293	446	0.004 **
Lepidoptera	Geometridae	*Melanolophia canadaria*	298	1.76	131	155	12	4.21	0.122	20	278	374.5	0.021 *

## Data Availability

The data that support the findings of this study will be available upon request.

## Supplementary Materials

The following supporting information can be downloaded at: https://www.mdpi.com/article/10.3390/plants11192596/s1, Table S1: A list of understory herbaceous species found during the flowering period of black cherry.
