# Supplementary material for: Effects of Forest Management on the Insect Assemblage of Black Cherry (Prunus serotina) in the Allegheny National Forest
Source: Plants (Basel). 2022 Oct 1;11(19):2596. doi: 10.3390/plants11192596 (PMC9572697; doi:10.3390/plants11192596)
Supplement: Supplementary file 1 [file plants-11-02596-s001.zip › plants-1903490-supplementary.pdf]

Table S1: A list of understory herbaceous species found during the flowering period of black cherry

Common name	Family	Genus	Species	Unmanaged stand	Seed-tree stand	Removal stand
Elderberry	Adoxaceae	*Sambucus*	*spp.*	*X*		
Staghorn Sumac	Anacardiaceae	*Rhus*	*typhina*	*X*		
Mountain Holly	Aquifoliaceae	*Ilex*	*montana*		*X*	
Jack-in-the-pulpit	Aracae	*Arisaema*	*triphyllum*	*X*		
Devil's walking stick	Araliaceae	*Aralia*	*spinosa*		*X*	
Dwarf Ginsing	Araliaceae	*Panax*	*trifolius*	*X*		
Canada Mayflower	Asparagaceae	*Maianthemum*	*canadense*	*X*		*X*
Solomon's seal	Asparagaceae	*Polygonatum*	*pubescens*	*X*		
Goldenrod	Asteraceae	*Solidago*	*spp.*		*X*	
Mayapple	Berberidaceae	*Podophyllum*	*peltatum*	*X*		
Black Birch	Betulaceae	*Betula*	*lenta*		*X*	*X*
Sessile Bellwort	Colchicaceae	*Uvularia*	*sessilifolia*	*X*		
Sedge	Cyperaceae	*Carex*	*debilis*	*X*	*X*	*X*
Pink Ladies Slipper	Cypripedioideae	*Cypripedium*	*acaule*	*X*		
Hay-scented fern	Dennstaedtiaceae	*Dennstaedtia*	*punctilobula*			*X*
Intermediate Wood Fern	Dryopteridaceae	*Dryopteris*	*intermedia*	*X*	*X*	*X*
American Beech	Fagacae	*Fagus*	*grandifolia*	*X*	*X*	*X*
Indian Cucumber Root	Liliaceae	*Medeola*	*virginiana*	*X*	*X*	
Trout Lily	Liliaceae	*Erythronium*	*americanum*	*X*		
Creeping Ground cedar	Lycopodiaceae	*Diphasiastrum*	*digatatum*	*X*		*X*
Ground Pine	Lycopodiaceae	*Lycopodium*	*dendroideum*		*X*	
Ground Pine	Lycopodiaceae	*Lycopodium*	*obscurum*	*X*	*X*	
Running Ground Cedar	Lycopodiaceae	*Diphasiastrum*	*digatatum*			*X*
Shining clubmoss	Lycopodiaceae	*Huperzia*	*lucidula*	*X*		
Cucumber Magnolia	Magnoliacae	*Magnolia*	*acuminata*	*X*	*X*	*X*
Tulip tree	Magnoliacae	*Liriodendron*	*tulipifera*	*X*	*X*	*X*
Painted Trillium	Melanthiaceae	*Trillium*	*undulatum*	*X*	*X*	
White trillium	Melanthiaceae	*Trillium*	*grandiflora*	*X*		
Green Ash	Oleaceae	*Fraxinus*	*americana*		*X*	
Beechdrops	Orobanchaceae	*Epifagus*	*virginiana*	*X*		
Cinnamon Fern	Osmundaceae	*Osmunda*	*cinnamomea*	*X*		
Wood Sorrel	Oxalidaceae	*Oxalis*	*montana*	*X*		
Eastern Hemlock	Pinaceae	*Tsuga*	*canadensis*	*X*		
Deer Tongue Grass	Poaceae	*Dichanthelium*	*clandestine*	*X*		*X*
Short Husk Grass	Poaceae	*Brachyelytrum*	*erectum*	*X*		*X*
Starflower	Primulaceae	*Trientilla*	*borealis*	*X*		
Blackberry	Rosaceae	*Rubus*	*allegheniensis*	*X*		*X*
Bristly Dewberry	Rosaceae	*Rubus*	*hispidus*	*X*		
Pin Cherry	Rosaceae	*Prunus*	*pensylvanica*		*X*	*X*
Raspberry	Rosaceae	*Rubus*	*idaeus*	*X*		
Serviceberry	Rosaceae	*Amelanchier*	*arborea*	*X*	*X*	
Black Cherry	Rosaceae	*Prunus*	*serotina*	*X*	*X*	*X*
Chokecherry	Rosaceae	*Prunus*	*virginiana*		*X*	
Partridgeberry	Rubiaceae	*Mitella*	*repens*	*X*		
Red Maple	Sapindaceae	*Acer*	*rubrum*	*X*	*X*	*X*
Striped Maple	Sapindaceae	*Acer*	*pensylvanicum*	*X*	*X*	
New York Fern	Thelypteridaceae	*Thelypteris*	*noveboracensis*	*X*		*X*
Round leaved violet	Violaceae	*Viola*	*rotundifolia*	*X*	*X*	
Sweet White Violet	Violaceae	*Viola*	*blanda*	*X*	*X*	*X*
